# Deep Learning-Assisted Early Detection of Skin Cancer from Dermoscopic Images in Underserved Clinical Settings

**DOI:** 10.3390/bioengineering13040456

**Published:** 2026-04-13

**Authors:** Anchal Kumari, Punam Rattan, Anand Kumar Shukla, Sita Rani, Aman Kataria, Hong Min, Taeho Kim

**Affiliations:** 1 School of Computer Application, Lovely Professional University, Jalandhar 144411, Punjab, India; anchalsharma897@gmail.com (A.K.); anand.shukla@lpu.co.in (A.K.S.); 2 Computer Science and Technology, Manav Rachna University, Faridabad 121004, Haryana, India; punamrattan@gmail.com; 3 Department of Computer Science and Engineering, Guru Nanak Dev Engineering College, Ludhiana 141006, Punjab, India; sitasaini80@gmail.com; 4 University Centre for Research and Development, Chandigarh University, Gharuan, Mohali 140413, Punjab, India; ammankataria@gmail.com; 5 School of Computing, Gachon University, Seongnam 13120, Republic of Korea; 6 Institute for Information & Communications Technology Planning & Evaluation (IITP), Daejeon 34000, Republic of Korea

**Keywords:** melanoma, skin cancer, clinic image dataset, Himachal Pradesh, ResNet50

## Abstract

Skin cancer is caused by aberrant cells that proliferate uncontrollably after unrepaired DNA damage results in mutations in the epidermis. The majority of skin cancer is caused by high UV exposure from the sun, tanning beds, or sunlamps. Due to sociocultural hurdles, limited access to specialized dermatological care, and low public knowledge, many nations, including India, have higher mortality rates and late-stage presentations. The unequal distribution of specialized dermatological treatments, particularly in rural and underdeveloped areas, makes detection and treatment more difficult. For skin cancer, one of the most prevalent malignancies with a high death rate, early detection is crucial. This study gathered 1200 dermoscopic images from two clinics in Himachal Pradesh in order to solve these problems. In order to automatically classify dermoscopic clinical images into melanoma and non-melanoma skin cancer categories, this study compares VGG16 with ResNet-50. Preprocessing, lesion segmentation, and classification are all part of the suggested approach. A collection of 1200 dermoscopic images with clinical annotations was used to improve the models. ResNet-50 outperformed VGG16 in tests, with 93% accuracy and 96% AUC-ROC as opposed to 89% and 94%, respectively. These results emphasize how crucial model selection and preprocessing are to diagnostic performance. Ensemble methods, multi-class classification, explainability integration, and clinical validation will be investigated in order to facilitate the implementation of AI-assisted dermatological diagnostic tools.

## 1. Introduction

The environment constantly affects one of the body’s most vital organs, the skin. Therefore, maintaining healthy skin and facilitating prompt assistance for issues are crucial [1]. Skin cancer originates from the uncontrolled proliferation of skin cells with defective DNA structure and is one of the most common diseases worldwide [2]. Early discovery of skin cancer allows for permanent therapy, making rapid diagnosis crucial for positive outcomes. However, the traditional method of detecting skin cancer by visually inspecting lesions with the naked eye reduces detection accuracy [3]. Due to modern technology breakthroughs, the utilization of deep learning techniques has become significant, and image-based classification methods for diagnosing skin lesions [4,5]. The skin is the body’s main defense against outside dangers, and it is very important for keeping the body healthy and well [6]. The largest organ of the human body serves as a protective barrier against harmful infections, physical trauma, and unfavorable environmental circumstances. The skin comprises three principal layers: the epidermis, dermis, and hypodermis [7,8]. Skin cancer is a major public health issue, influenced by factors such as prolonged sun exposure, genetic predisposition, and environmental triggers. Forecasts for 2024 in the United States predict 108,270 new skin cancer cases, leading to 13,120 deaths [9].

As with all forms of cancer, early detection is crucial for skin cancer. Although skin cancer detection has become easier thanks to deep learning, there are still certain challenges. The accuracy of feature extraction and classification can be adversely affected by defects in dermoscopic images, such as hair obstruction, uneven illumination, and background noise [10]. A lot of current research either does not do enough preprocessing or relies on publicly available datasets that may not adequately reflect real clinical circumstances, especially in locations with limited resources [11,12]. Additionally, similar evaluations of CNN architectures using clinically acquired datasets are limited, hindering the selection of suitable models for reliable melanoma diagnosis [13]. These shortcomings reduce the clinical significance of AI-based diagnostic instruments. This paper introduces a comprehensive DL architecture for the classification of skin lesions using the CSI dataset. The framework integrates optimal image preprocessing techniques, including scaling, morphology-based hair removal, and noise reduction, to enhance skin lesion visibility and minimize artifacts in the skin image.

The work was performed with a comprehensive comparative analysis of two widely used pre-trained CNN architectures, VGG16 and ResNet-50, using transfer learning and fine-tuning methodologies on a meticulously selected CSI dataset of 1200 dermoscopic images collected from clinics in Himachal Pradesh and Ludhiana, India. This paper conducts a systematic comparison of multiple transfer learning architectures, VGG16 and ResNet-50, using a unified experimental framework with uniform dataset splitting, optimization settings, and evaluation criteria.

We utilize performance metrics such as accuracy, precision, recall, F1-score, and AUC-ROC to evaluate the model’s efficacy. Gradient-weighted Class Activation Mapping (Grad-CAM) is employed to enhance model interpretability by highlighting regions significant for diagnosis. The proposed technique aims to identify an effective and clinically reliable deep learning model for the early detection of melanoma.

### 1.1. Contribution of This Research

- This paper provides several significant additions to the field of automated skin cancer diagnosis utilizing deep learning approaches, particularly in the classification of dermoscopic images into melanoma and non-melanoma categories.
- This study introduces a comprehensive image preprocessing methodology that effectively addresses common noise factors in dermoscopic images, including hair artifacts and corner black pixels. By employing a morphology-based hair removal technique combined with median filtering and cropping, the preprocessing stage enhances lesion visibility and reduces background noise, thereby improving the quality of input data for subsequent segmentation and classification tasks.
- The proposed method incorporates an accurate lesion segmentation phase that isolates the skin lesion from surrounding healthy tissue. This paper presents a rigorous comparative analysis of two prominent convolutional neural network architectures, VGG16 and ResNet-50, employing transfer learning and fine-tuning strategies on a curated clinical dermoscopic dataset.
- This paper assesses deep learning models utilizing dermoscopic images gathered from regional clinical settings in Himachal Pradesh and Ludhiana, India, in contrast to many other studies that only use big public datasets. This dataset offers important insights into the application of AI in underprivileged healthcare settings by capturing actual issues, including imaging variability, class imbalance, and resource scarcity.
- Grad-CAM visualization was used to highlight lesion regions, influencing the model’s decisions regarding interpretability. This approach provides dermatologists with visual explanations of predictions.
- This work offers a methodical evaluation of deep learning models using uncurated clinical photos, showcasing resilience in real-world scenarios not included in benchmark datasets.

### 1.2. Related Work

In recent years, a number of studies have looked at various computer-aided methods for detecting skin cancer. In [14], the authors looked at how well ResNet-50v2, DenseNet201, GoogleNet, MobileNetv2, VGG16, Resnet152v2, Xception, and VGG19 could tell if a skin spot was a melanoma. There were three groups in the dataset: training (80%), validation (10%), and testing (10%). The most accurate model was GoogleNet, which got 76.08% of the classifications right. The researchers in [15] used a region-based and mask convolutional neural network to identify the diseased area. They then used Resnet152 to classify the lesions as either benign or malignant. The performance of automatic region-of-interest extraction in dermoscopic images is greatly enhanced by removing pixels from the picture that do not contain information about the tumor. A technique was introduced by [16] to detect both benign and malignant cancer types with ResNet-50, Inceptionv3, and ResNet Inception deep learning models. The suggested approach enhances the brightness of lesion pictures and reduces noise using image enhancement techniques, attaining ideal results with an accuracy of 0.8576 when utilizing the Inceptionv3 model. The aim of this work is to aggregate the preprocessing approaches relevant to skin cancer images. The study by [17] provides a significant basis for researchers in the domain of automated skin cancer diagnosis. It emphasizes the methodology; datasets; performance indicators, including accuracy and precision; distinctive methodological contributions; and limits recognized by each study. The study by [18] presents an improved method for enhancing skin image classification to remove sparse noise. The study by [19] combined a genetic algorithm (GA) with the Alternating Direction Method of Multipliers (ADMM). That study used the DenseNet method for the classification of the optimized sparse matrix. Contrastive learning focuses on the use of genome methods, physiological signals, medical imaging, and EHRs in medicine to develop advanced systems for healthcare. An enhanced technique was developed in [20] by combining an improved sparse autoencoder (SAE) for feature learning with a Softmax regression classifier for disease classification. Instead of using activations, the SAE regularizes weights, which improves performance. The approach outperformed existing machine learning methods with accuracies of 91%, 97%, and 98% on datasets for heart disease, cervical cancer, and chronic kidney disease, respectively. Using a sizable dataset, [21] created hybrid ensemble models and improved machine learning models for coronary heart disease (CHD) prediction. They achieve excellent accuracy, sensitivity, and specificity by comparing upgraded versions and ensemble techniques with baseline models such as decision trees and support vector machines. Scalability and robustness are highlighted in that study, especially in situations with limited resources. Using SHapley Additive exPlanations (SHAPs) to explain model predictions, their research proposes an interpretable machine learning method for diagnosing hepatitis B. In [22], bilirubin was identified as a critical factor in predicting patient mortality after evaluating several machine learning algorithms. The goal of that project was to increase model openness and help medical professionals comprehend machine learning choices.

A number of deep learning models, including VGG16, ResNet-50, Xception, DenseNet169, and MobileNetv2, were compared for performance in this study. Because of their unique designs and proven efficacy in image classification tasks, we selected VGG16 and ResNet-50 for in-depth examination in this study. Despite being taken into consideration, models like MobileNetv2 were not included in the primary findings because of their poorer dataset performance, which was demonstrated by early tests.

### 1.3. Paper Outline

The outline of this paper is as follows: Section 2 describes this study’s proposed methodology. Section 3 describes the methods and feature extraction results using CNN pre-trained models. In Section 4, the experimental results are fully analyzed, comparing pre-trained CNN models. In addition, that section summarizes the findings, highlighting the ResNet-50 model’s higher performance in binary melanoma and non-melanoma lesion categorization. Finally, Section 5 suggests clinical validation studies, comprehension framework integration, multi-class classification, ensemble modeling, and other research directions to improve AI-driven skin cancer diagnostic tools.

## 2. Proposed Methodology

**Figure 1:**
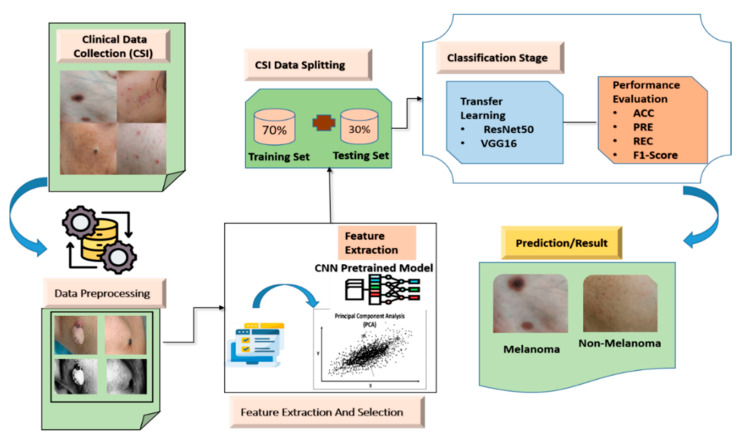
The proposed dermoscopy-driven deep learning framework for the classification of melanoma and non-melanoma skin lesions.

The proposed approaches to dermatological condition classification are outlined and evaluated in this part. The entire procedure consists of the following components: the initial step involves picture preprocessing, followed by image segmentation, then feature extraction, concluding with classification. Figure 1 illustrates the proposed dermoscopy-driven deep learning framework for the classification of melanoma and non-melanoma skin lesions.

### 2.1. Dataset Overview

A total of 1200 photos from the Ziva Skin & Hair Clinic in Hamirpur, Himachal Pradesh, and Sparsh Aesthetics in Ludhiana, with various attributes, such as the patient’s gender, the affected area, symptoms, and the name of the skin illness, were used to assess the suggested techniques. The specifications of the clinical data are shown in Table 1.

### 2.2. Image Preprocessing

These images exemplify key morphological features such as asymmetry, border irregularity, color variation, and texture heterogeneity, which are critical for accurate skin cancer diagnosis [23]. Image preprocessing is the process of converting an image into a more suitable and useful form. Skin scans can show distortion, interference, or superfluous hair. The quality of the photographs defines the effectiveness of an image processing system. Processing reduces complexity, improves accuracy, and raises image quality for subsequent processing. The preprocessing steps include image resizing, hair removal and noise elimination.

Images of varying dimensions do not consistently exhibit an equivalent number of characteristics. Therefore, the input images are scaled to mitigate this issue. This diminishes processing duration and improves the system’s overall efficacy. In this investigation, we resized all input images to dimensions of 224 × 224.

**Figure 2:**
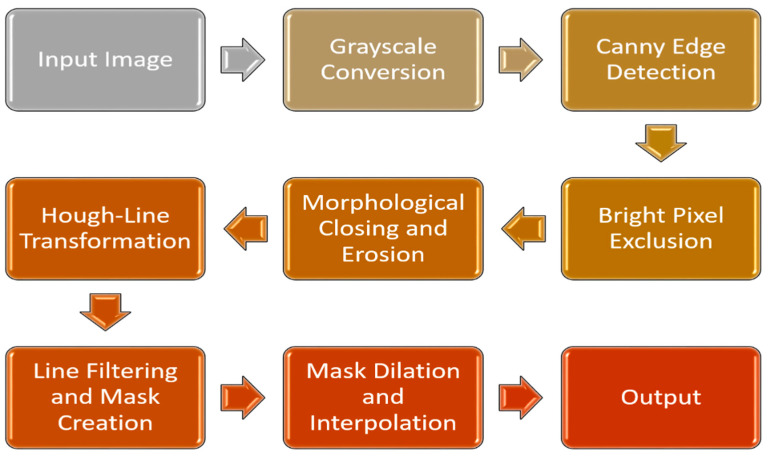
Various phases of image preprocessing.

The stages of preprocessing and feature extraction are described in Figure 2. Initially, the picture was transformed to grayscale, eliminating color information that might obstruct edge detection. The Canny edge detector was utilized to identify significant variations in brightness that indicate edges in the hair. Highly bright pixels were excluded due to their low probability of representing hair. A morphological closing procedure was performed to improve edge refinement, smooth edges and close tiny gaps. Erosion was subsequently utilized to remove solitary, noisy pixels that could be mistakenly identified as edges. The probabilistic Hough Line Transform was subsequently employed to detect lines inside the improved edges. Since they are unlikely to resemble hair, lines that are too short or long have been removed. The pixels in the dilated mask have been interpolated with the median value of the neighboring pixels, thus filling gaps in the hair and producing a more polished outcome.

### 2.3. Feature Extraction and Selection by Using Traditional Approaches

We used gradient-based descriptors, such as Canny edge detection and HOG, in conjunction with texture and form characteristics, including area, perimeter, and contour anomalies, for feature extraction. These techniques are essential for precisely identifying and evaluating textural characteristics and lesion boundaries in dermoscopic images. Using the standard procedure for extraction and selection, the following properties have been recorded in Table 2.

HOG is the descriptor for forms and textural aspects that depend on gradient orientations and magnitudes; it is derived from grayscale pictures and does not directly employ color data.

**Figure 3:**
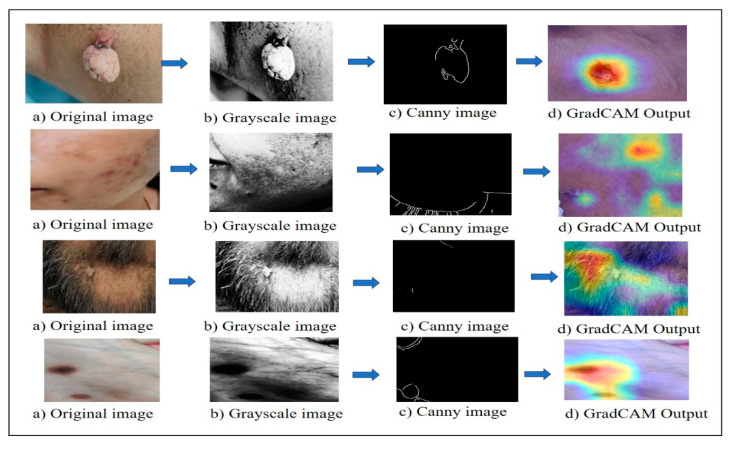
Visualization of the skin lesion analysis pipeline for image preprocessing and model interpretability.

The Canny edge detection technique is used to find edge characteristics in pictures of skin. This approach works well for finding sharp intensity gradients, which makes it possible to precisely locate the edges of lesions by getting rid of noise and linking edge segments. The Canny method is useful for finding clinically important edges in dermatological images because it uses several steps, including calculating gradients, suppressing non-maxima, and using hysteresis thresholding. Figure 3 shows a visualization of the skin lesion analysis pipeline for image preprocessing and model interpretability. Skin lesion images are processed through four stages: (a) the original clinical image; (b) the corresponding grayscale conversion used to enhance structural details; (c) edge detection using the Canny algorithm to define lesion boundaries and textures; and (d) Grad-CAM (Gradient-weighted Class Activation Mapping) heat maps superimposed on the original image. Grad-CAM creates heat maps that reveal the model’s viewing location in the image while classifying. This is accomplished by utilizing the gradients that flow into the network’s last convolutional layers. A coarse localization map was computed using gradients, which highlights areas of increased decision importance. The right panel shows a high-resolution microscopic picture of the skin with the heat map superimposed on top; this allows one to see exactly which parts of the picture were crucial to the model’s predictions. By incorporating Grad-CAM, the suggested method improves both visibility and error analysis. Additional investigation into misclassified cases, which might be features inside or unrelated to the region of interest, can be carried out using heat maps. A dimensionality reduction technique called principal component analysis (PCA) turns a set of possibly correlated variables into a set of linearly independent variables.

### 2.4. Using Grad-CAM to Make AI Understandable

For clinical decision-support systems that use deep learning models, explainability is very important, especially when they are used for high-risk tasks like finding skin cancer. To improve transparency and therapeutic trust, this work uses Gradient-weighted Class Activation Mapping (Grad-CAM) as an explainable AI tool for visually interpreting model predictions. Grad-CAM works by calculating the gradients of the projected class score in relation to the feature maps of the last convolutional layer. This makes a rough localization map that shows the parts of the picture that had the biggest effect on the classification decision. When looking at dermoscopic images, these heat maps always highlight important clinical lesion features such as asymmetry, border abnormalities, texture heterogeneity, and color variations. By putting the Grad-CAM heat maps on top of the original dermoscopic pictures, physicians can see where the model is focusing its attention.

We adjusted for the dataset’s 5:1 class imbalance using a weighted binary cross-entropy loss function. Specifically, the weight of the melanoma class was increased to emphasize its detection. In order to mitigate this problem, the model was trained using a weighted binary cross-entropy loss function, which penalizes misclassification of melanoma images more severely. These class weights were computed by Equation (1):

(1) wc=NC∗nc

where w_c_ is the class weight, N is the number of samples, C is the number of classes, and n_c_ is the number of sample classes. After that, the resulting weight for melanoma for the 200 samples is 3.0, and for non-melanoma, it is 0.6.

We conducted additional ablation trials to verify the effectiveness of this alteration, as shown below in Table 3.

The ablation findings show that class weighting increases melanoma sensitivity (recall) from 0.75 to 0.93, a substantial improvement. This demonstrates that the model significantly improves its ability to identify cases of minority-class melanoma, which is crucial in clinical diagnostics where false negatives need to be reduced. Overall, the weighted loss increases the AUC from 0.88 to 0.96, indicating improved robustness and class separability.

## 3. Method

### 3.1. Experimental Framework

The experimental framework was executed in a regulated and replicable computational setting utilizing Python (version 3.9) as the primary programming language, with deep learning models created and trained through TensorFlow 2.x and the Keras API. OpenCV and scikit-image were used for picture preparation tasks like scaling, converting to grayscale, removing hair with morphological techniques, finding edges, and reducing noise. NumPy and Pandas were used for numerical calculations and working with datasets. All of the tests were done on a workstation with an NVIDIA GPU, CUDA toolkit, cuDNN acceleration, 16 GB of RAM, and SSD storage. This made training and inference quick and easy.

### 3.2. Configuration for Model Experiment

For each experiment, 80% of the dataset was used for training, 10% for validation, and 10% for testing. To ensure that the class distribution in each split was consistent and to prevent any potential data leakage, stratified sampling was employed. Our approach ensured that each model was trained on a representative sample of the entire dataset by maintaining a constant ratio of melanoma to non-melanoma photos across all splits. A fixed random seed of 42 was applied to the same data split in subsequent tests. The Adam optimizer was used to train all models, ensuring consistent convergence throughout. Parameters of Adam optimizer: learning rate = 0.0001, β1 is 0.9, β2 is 0.999, and weight decay is 1 × 10^−5^.

The model was trained using a batch size of 32 for 50 epochs. For completely connected layers, the dropout rate was 0.5. Early halting was started when validation loss did not improve for five consecutive epochs. Every ten epochs, the learning rate scheduler used a 0.0001 initial learning rate with a 0.9 factor of exponential decay. This configuration was used for all models, including ResNet-50, VGG16, Xception, DenseNet169, and MobileNetv2, in order to provide consistent training settings across testing.

Training, validation, and evaluation sets were kept strictly apart throughout the process to ensure experimental rigor and to avoid data leaking. Normalization and augmentation, two preprocessing techniques that potentially cause data-dependent bias, were only used after the dataset was split. Only the training set was subjected to data augmentation (such as rotation, flipping, and brightness alteration); the validation and evaluation sets were kept entirely hidden and unchanged, with the exception of deterministic scaling and normalization. To further ensure independence between training and evaluation data, no pictures from identical patients or acquisition sessions were broken up over several splits. The consistency of the data splitting procedure was further ensured by the use of a consistent random seed.

### 3.3. Implementation of CNN Pre-Trained Model for Feature Extraction (ResNet-50 Model, VGG16 Model, Xception, DenseNet169 and MobileNetv2)

ResNet-50, a 50-layer deep residual network pre-trained on the ImageNet dataset, functions as an effective feature extractor for clinical images by transfer learning [24]. By eliminating the original classification head and employing the convolutional base, ResNet-50 acquires intricate hierarchical representations encompassing texture, shape, and color information inherent to medical imaging modalities.

The deep convolutional neural network architecture VGG16 [25] is distinguished by its basic, yet powerful design and consistent use of modest 3 × 3 convolutional filters. Using parallel convolutional filters of different sizes inside its Inception modules, the inception architecture, also known as GoogLeNet, allows for multi-scale feature extraction that captures various spatial patterns. Presented on the ImageNet dataset, the Inception model removes the last classification layers and uses the convolutional base as a feature extractor to efficiently transfer learnt visual features to clinical image analysis. Rich, multi-scale feature representations produced by this method capture intricate textural and structural information, qualities vital for identifying small disease changes.

By substituting depth-wise separable convolutions for conventional inception modules, which effectively capture spatial and channel-wise correlations and, therefore, lower model complexity, the Xception architecture expands the Inception model [26]. Xception creates detailed feature embedding on a convolutional basis and removes the top classification layers that enhance classification tasks in medical imaging, especially when training data is limited.

The design of DenseNet169 [27] is a densely linked convolutional neural network, which allows for enhanced information flow and gradient propagation by connecting each layer to every other layer in a feed-forward fashion. Originally trained on the ImageNet dataset, DenseNet169 removes its final classification layers and uses dense connectivity to capture complex textures and form patterns inside medical images, therefore acting as a potent feature extractor for clinical images.

MobileNetv2 [28] is a lightweight convolutional neural network architecture designed for efficient inference on resource-constrained smartphones using inverted residuals and linear bottlenecks to preserve representational power with fewer parameters. Pre-trained on the ImageNet dataset, MobileNetV2 performs as an effective feature extractor for clinical images by truncating the last classification layers and exploiting its convolutional basis to gather prominent hierarchical properties related to texture, shape, and color. Especially helpful when using clinical imaging models in limited-compute situations, this model’s efficiency allows for fast extraction of deep representations appropriate for downstream classification tasks.

### 3.4. Mathematical Approach

Automated skin lesion diagnosis is reframed as a supervised binary classification problem in the suggested framework in Equation (2):

(2) $$D=\left(x_{i},y_{i}\right)y_{i=1}^{N}$$

where D denotes the dataset, $x_{i}\in \;R^{224*224*3}$ represents a preprocessed dermoscopic image, and $y_{i}\in \left\{0,1\right\}$ corresponds to non-melanoma skin cancer (NMSC) and melanoma (MEL).

Image preprocessing is conceptualized as a composite change in Equation (3).

(3) $$\emptyset \left(x\right)=\emptyset_{s}[\emptyset_{h}\left({(\emptyset }_{r}\left(x\right)\right)]$$

where $\emptyset_{s}$ pertains to lesion segmentation, $\emptyset_{h}$ indicates morphological hair and noise reduction, and $\emptyset_{r}$ signifies resizing. This modification diminishes noise variance and amplifies lesion-specific information.

In conventional analysis, texture and form characteristics are derived using statistical descriptors such as the Gray-Level Co-occurrence Matrix.

DL models acquire a nonlinear correspondence in Equation (4).

(4) $$f_{\theta }:x\to \left[0,1\right]$$

This equation is parameterized by θ, transforming the input x into a continuous value in the interval [0, 1]. Convolutional layers generate hierarchical feature representations in Equation (5).

(5) $$h^{\left(l\right)}=\sigma \left(w^{\left(l\right)}*h^{\left(l-1\right)}+b^{\left(l\right)}\right)$$

The activation at layer *l* is represented by $h^{\left(l\right)}$, the weight matrix is $w^{\left(l\right)}$, the bias vector is $b^{\left(l\right)}$, and σ is a nonlinear activation function.

ResNet-50 employs residual connections of form in Equation (6), where $F\left(h^{\left(l\right)}\right)\;$ residual mapping is typically built from many convolutional layers.

(6) $$h^{\left(l+1\right)}=h^{\left(l\right)}+F\left(h^{\left(l\right)}\right)$$

This equation can mitigate vanishing gradients and promote deeper, more intricate feature learning relative to VGG16. Training reduces binary cross-entropy loss in Equation (7):

(7) $$L=-\frac{1}{N}\sum_{i=1}^{n}[y_{i}log{\hat{y}}_{l}+\left(1-y_{i}\right)log\left(1-{\hat{y}}_{l}\right)]$$

where *L is the binary cross-entropy loss value for the specified dataset*, and *N is* the dataset’s entire sample. $y_{i}$ is the exact ground truth label for the ith sample. In binary classification, this is often 0 or 1. ${\hat{y}}_{l}$ is the predicted probability for the ith sample, which belongs to the positive class (class 1). This is often the result of a sigmoid activation function. The model produces an estimated posterior probability of P(y = 1|x), aiding in threshold-based clinical decision-making. Evaluation metrics include accuracy, precision, recall, F1-score, and AUC-ROC to gauge classification efficacy. The superior AUC and accuracy achieved by ResNet-50 illustrate its advanced capacity to comprehend complex, nonlinear decision limits crucial for reliable melanoma detection.

To ensure reproducibility and a fair comparison across various model architectures, all experiments were carried out using the same preprocessing pipeline, training schedule, and evaluation protocol.

## 4. Results and Discussions

### 4.1. Clinic Skin Cancer Images Using ResNet-50

**Figure 4:**
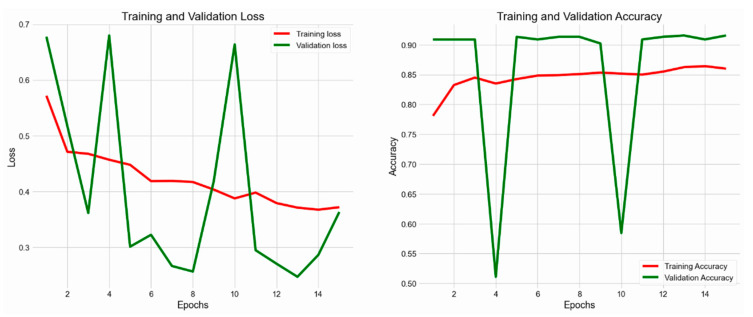
Training and validation accuracy or loss of ResNet-50 model.

This research employed ResNet-50 using transfer learning, utilizing weights pre-trained on the ImageNet dataset. The convolutional basis layers were refined with a specialized dermoscopic dataset to tailor the model for skin cancer detection. Image preprocessing standardized input dimensions to 224 × 224 pixels, conforming to the specifications of ResNet-50. The model utilized the Adam optimizer, along with a learning rate scheduler and a binary cross-entropy loss function. The experimental evaluation revealed that ResNet-50 achieved an accuracy of 93% in classifying MEL and non-melanoma skin cancer (NMSC), demonstrating substantial discriminatory capability, as well as training and validation accuracy or loss, as shown in Figure 4.

### 4.2. Clinic Skin Cancer Images Using VGG16

**Figure 5:**
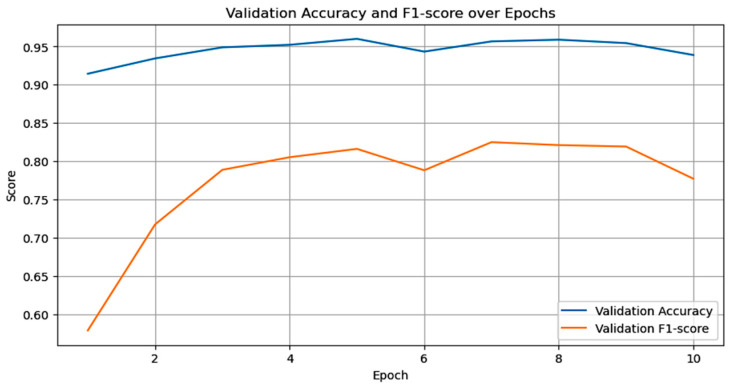
The accuracy and loss of the VGG16 Model during training and validation.

The dermoscopic images that were input were normalized and scaled to 224 × 224 pixels. The goal of data augmentation was to enhance generalizability by increasing the size of the training dataset. Zooming, brightness changes, horizontal and vertical flips, and random rotations were all strategies used for augmentation. The Adam optimizer was used to train the model, and the starting learning rate was set to 0.0001. The loss function utilized was binary cross-entropy. The three subsets of the dataset were as follows: 80% for training, 10% for validation, and 10% for testing. In order to avoid overfitting and to keep the top-performing model, we used early halting and model checkpoints. Figure 5 shows the accuracy or loss of the VGG16 model during training and validation.

**Figure 6:**
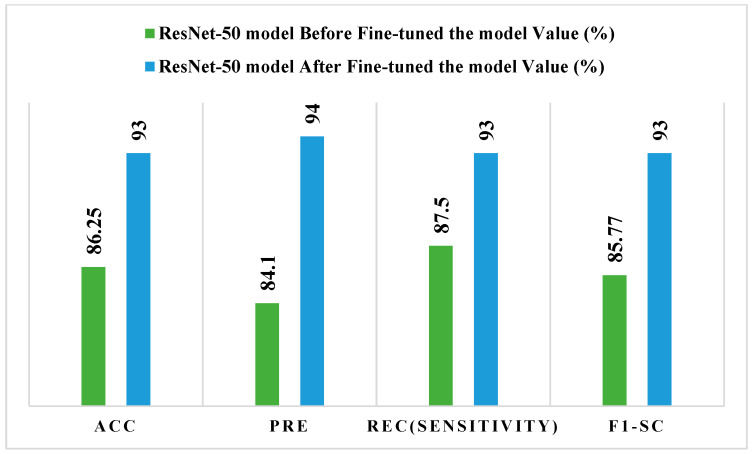
Performance test of the ResNet50 model before and after fine-tuning.

Figure 6 depicts the ResNet-50 model before and after conventional performance tests, which were used to fine-tune it. Precision improves from 84.10% to 94%, recall (sensitivity) from 87.50% to 93%, F1-score from 85.77% to 93%, and accuracy from 86.25% to 93% following fine-tuning. These results demonstrate that fine-tuning considerably improves the ResNet-50 model’s discriminative capabilities and classification resilience.

**Figure 7:**
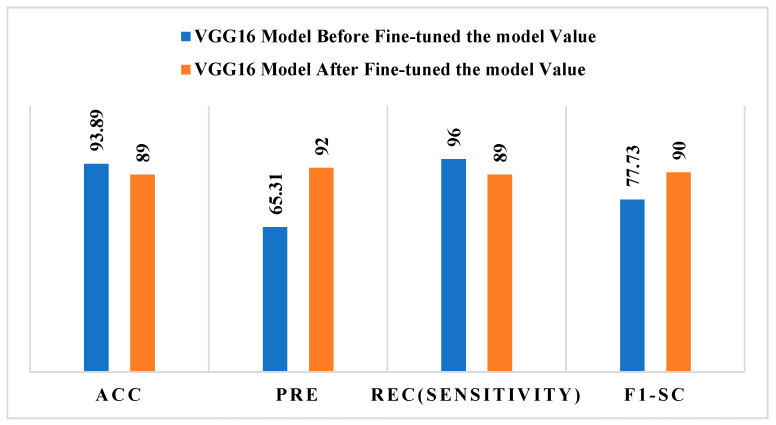
Performance test of the VGG16 model before and after fine-tuning.

Figure 7 shows how the VGG16 model performed before and after fine-tuning, using conventional evaluation measures to compare the two. Before fine-tuning, the model had a high accuracy (93.89%) and recall (96%) but a poor precision (65.31%) and F1-score (77.73%). This means that the model was making predictions that were not evenly distributed across classes. After fine-tuning, precision went up a lot, to 92%, and F1-score went up to 90%. However, accuracy and recall went down a little, to 89%.

These results indicate that fine-tuning improves the model’s accuracy and overall balance between sensitivity and specificity, resulting in more dependable melanoma categorization. After fine-tuning, a side-by-side comparison of the ResNet-50 and VGG16 models shows different performance patterns. The ResNet-50 model shows steady and constant progress across all assessment criteria, with accuracy, precision, recall, and F1-score scores of about 93–94%. This means that the model is better at representing features and classifying data. On the other hand, the VGG16 model shows a trade-off after fine-tuning, with big improvements in precision and F1-score, which means better prediction balance, but a small drop in accuracy and recall.

In addition to its deeper architecture and residual connections, ResNet-50’s improved performance can be attributed to its capacity to capture complicated aspects in the dataset. Our morphology-based preprocessing workflow produces high-quality dermoscopic pictures that improve feature visibility and allow the model to extract more discriminative and detailed features. Additionally, the model is able to generalize better on the minority class (melanoma) without overfitting to the majority class (non-melanoma) thanks to ResNet-50’s residual connections, which assist in alleviating problems caused by the dataset’s class imbalance.

### 4.3. Comparative Analysis of ResNet-50 and VGG16 Models

**Figure 8:**
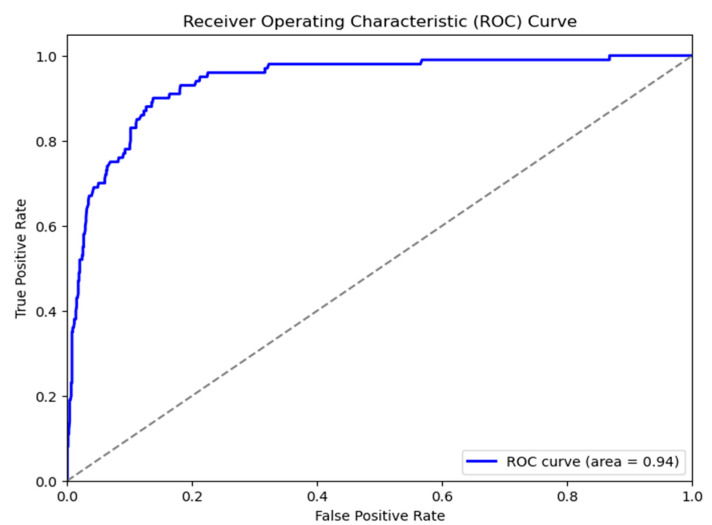
ROC curve for CSI cancer detection by implementing VGG16 model.

**Figure 9:**
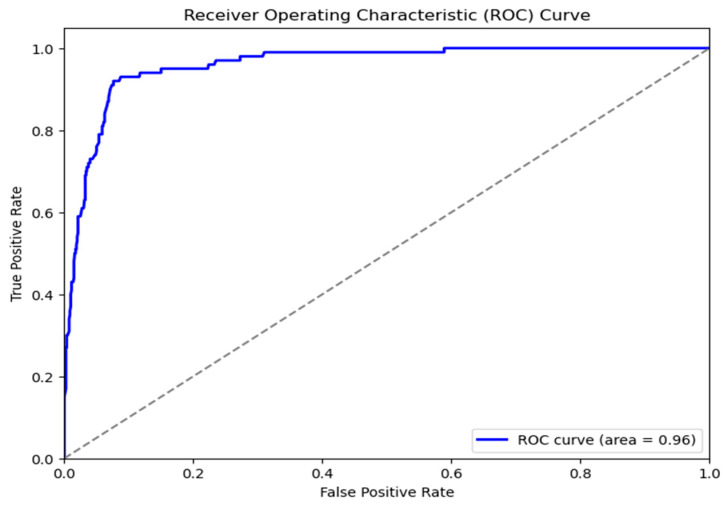
ROC curve for CSI cancer detection by implementing ResNet50 model.

Automated skin cancer identification using dermoscopic images is the subject of this study, which compares and contrasts two well-known deep convolutional neural network designs, ResNet-50 and VGG16. The ResNet-50 model achieved an AUC of 0.96, indicating an exceptional capacity to distinguish between positive and negative classifications. The curve demonstrates a rapid increase in TPR with few variations in FPR, suggesting robust predictive effectiveness with decreased false positives. The VGG16 model demonstrated somewhat diminished, albeit strong, performance with an AUC of 0.94. The ROC curve has a similar pattern but indicates a slightly higher rate of false positives compared to the true-positive rate when juxtaposed with ResNet-50. The AUC difference (0.96 vs. 0.94), illustrated in Figure 8 and Figure 9, demonstrates that ResNet-50 outperforms VGG16 in this classification task, presumably owing to its deeper architecture and the integration of residual connections, which improve gradient flow and facilitate the learning of more complex features.

The testing results demonstrate that ResNet-50 surpassed VGG16, attaining an accuracy of 93% in contrast to 89%. This outcome may first seem paradoxical, given ResNet-50’s sophisticated residual learning framework, intended to mitigate vanishing gradients in deeper architectures. The increased parameter count in ResNet-50 enhances its representational ability, perhaps aiding in the capture of complex morphological changes in skin lesions in the specified dataset.

**Figure 10:**
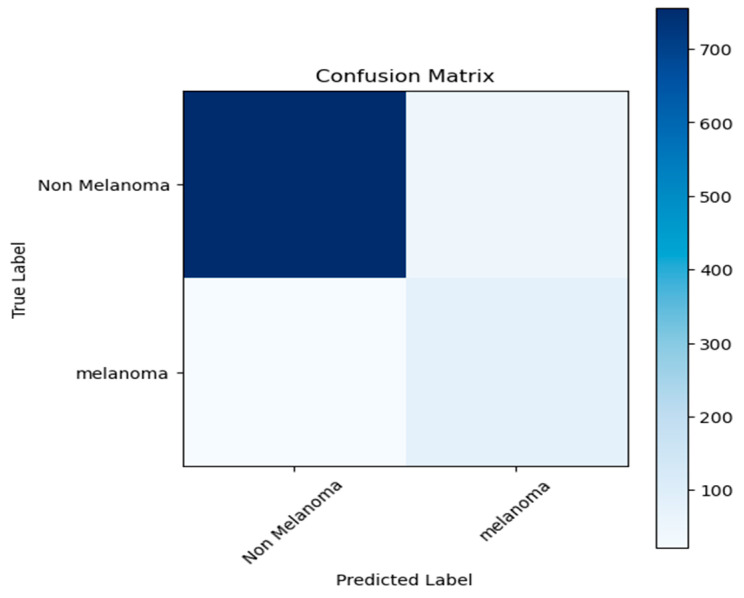
A confusion matrix for melanoma identification.

The confusion matrix in Figure 10 shows that the proposed model is very good at finding non-melanoma lesions since it has a high true-negative rate. The algorithm properly identifies the majority of melanoma cases; nonetheless, a portion of melanoma samples continues to be misclassified as non-melanoma, which is clinically important. This shows that the model needs to be improved even more to reduce false negatives and to make melanoma detection more reliable.

**Figure 11:**
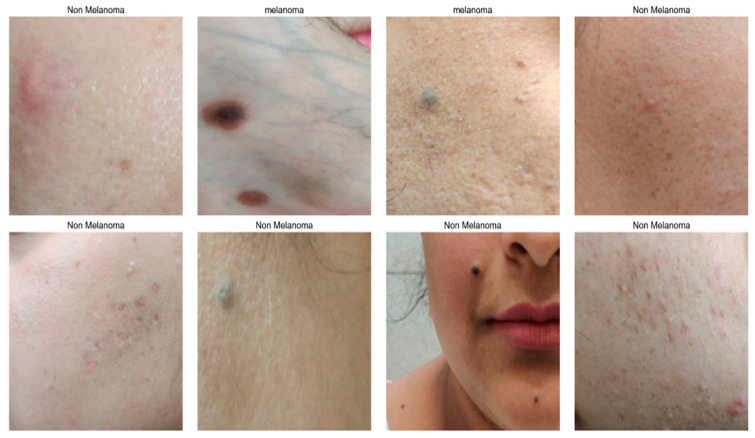
Classification of skin cancer into two categories.

Figure 11 illustrates the different skin cancer types as two categories: melanoma and non-melanoma.

### 4.4. Comparison with Existing Study

Table 4 displays the dataset used in this research, technique details, and the results of the comparison of the proposed models with existing model results.

**Table 4:** A comparative analysis of the proposed model with existing models.

	Ref.	Used Dataset	Techniques Used	Accuracy	Precision	Recall	F1-Score
	[16]	HAM10000	Modified ResNet-50	86.00%	84.00%	86.00%	86.00%
	[29]	ISIC 2015, ISIC 2019	CNN pre-trained model	88.83%	91.07%	87.68%	89.32%
EXISTING METHOD	[30]	HAM10000	ResNet, InceptionV3, and ResNet Inception	85.7%	83.00%	86.00%	84.00%
	[31]	Kaggle Datasets	AlexNet, MobileNet, ResNet, VGG16, and VGG19	84.94%	79.29%	1.00%	88.45%
	[32]	PH2, ISBI2016, and ISIC2017	ResNet50	86.5%	87.01%	85.57%	86.28%
	[33]	Kaggle and ISIC Datasets	EfficientNetB3, MobileNetV2	90.7%	95.00%	97.00%	95.00%
PROPOSED METHOD	RESNET50	Clinical Image Data (CSI)	ResNet50	93.00%	94.00%	93.00%	93.00%
	VGG16	Clinical Image Data (CSI)	VGG16	89.00%	92.00%	89.00%	90.00%

### 4.5. Critical Evaluation of Existing Work and Progressive Contributions

Although Table 3 offers a quantitative comparison, a more thorough analysis reveals a number of significant differences between the suggested study and the body of the current literature. The majority of earlier studies depend on publicly available benchmark datasets (such as ISIC and HAM10000), which are usually carefully selected and recorded under controlled circumstances. This study, on the other hand, focuses on actual clinical data from underprivileged settings, which presents difficulties, including noise, class imbalance, and acquisition condition unpredictability. As a result, the problem setting is more typical of real-world deployment situations.

Despite the model’s overall good performance, several melanoma samples that were misidentified pointed to significant areas that needed work. For instance, the paucity of training data for these specific cases may have contributed to the model’s frequent misclassification of melanomas with small lesions or uneven margins. When we looked at the inaccurately classified data, we saw that the model struggled with images that had background noise or hair artifacts, despite our preparation efforts. These misclassifications suggest that the model would benefit from more training with more challenging instances, particularly small and atypical melanomas, which are underrepresented in the dataset.

Additionally, Grad-CAM is incorporated into this work as a validation method to evaluate clinical feature alignment, guaranteeing that model choices agree with medically significant traits such as asymmetry and uneven borders. This closes the gap that has been understudied in previous research between algorithmic predictions and physician interpretability. This study stresses a system-level adaptation of current designs (ResNet-50 and VGG16) under a unified and regulated experimental framework, in contrast to works that concentrate on suggesting new topologies.

### 4.6. Effectiveness and Clinical Significance

The proposed transfer learning system demonstrated superior performance in binary skin cancer classification, with ResNet-50 exceeding VGG16 in accuracy and AUC-ROC metrics. The enhanced efficacy of ResNet-50 can be ascribed to its deeper architecture and residual connections, facilitating the proficient acquisition of intricate lesion characteristics. Elevated sensitivity is essential in melanoma diagnosis owing to the grave repercussions of overlooked diagnoses. Employing a weighted loss function enhances melanoma detection while preserving an equitable balance with specificity. Consistently elevated AUC-ROC values signify dependable differentiation across choice thresholds.

Based on their capacity to catch intricate details in dermatological images, we concentrated our tests on two well-known models, VGG16 and ResNet-50. Based on an earlier study, several models were excluded from the final comparative analysis because of their less-than-ideal performance. We prioritized VGG16 and ResNet-50 for further investigation since these models showed either slower convergence or worse AUC-ROC scores in early trials.

Because Grad-CAM interpretability was incorporated, clinicians were better able to understand and accept the model’s suggestions, especially in areas where they lacked knowledge. By highlighting areas of the image that most influenced the model’s classification decision, Grad-CAM helps ensure that the model’s predictions are consistent with clinically significant features, such as asymmetry and border irregularities. This increases confidence in AI-assisted diagnosis, especially in impoverished areas where dermatological expertise is scarce.

An optimization method based on data augmentation and decision threshold adjustment was used to reduce false negative predictions. In order to improve model sensitivity and give priority to the identification of possibly malignant lesions, the melanoma classification threshold was first modified. Second, during training, additional augmentation methods that highlighted lesion contrast and scale differences were used.

### 4.7. Considerations for Edge Inference and Deployment Feasibility

We assessed the computational viability of the suggested framework for deployment in low-resource clinical environments based on model complexity, input size, and inference characteristics noted during experimentation, even though this study did not include full hardware-level benchmarking on dedicated edge devices. The suggested models (ResNet-50 and VGG16) use photos with a resolution of 224 × 224, and inference was carried out effectively on a workstation with a GPU, showing no signs of processing bottlenecks. Both architectures can be modified for deployment on resource-constrained devices using common optimization techniques like model quantization and pruning because they are widely supported by lightweight inference frameworks like TensorFlow Runtime.

### 4.8. Tuning and Optimizing Hyperparameters

The model’s hyperparameters were optimized to enhance its functionality, prevent overfitting, and increase its generalizability. Through a comprehensive evaluation on the validation set, we successfully optimized critical hyperparameters such as learning rate, batch size, the number of trainable layers, dropout rate, and class weight values. We maintained a consistent convergence by meticulously adjusting the learning rate. If one wants to speed up training while keeping losses modest, the best starting value is 0.0001. The GPU’s memory and the model’s convergence speed were the determining factors in the batch size that we used to keep the gradient updates within budget. We used dropout regularization on the completely connected layers to prevent the model from fitting too well. In order for the models to be able to process dermoscopic data that is particular to a certain field without affecting previously trained representations, we intentionally altered the deeper convolutional layers. We adjusted the class weights in the weighted binary cross-entropy loss function to make melanoma more sensitive while keeping the overall specificity the same in order to fix the imbalance between classes. To prevent performance from deteriorating further, we also used early halting. Because all of the tested deep learning architectures performed adequately and fairly, this configuration of hyperparameter tuning made melanoma detection easy.

A significant issue with our model was the frequency of false negatives, in which cases of melanoma were mistakenly classified as non-melanoma. In clinical settings, where missed diagnoses can lead to treatment delays, these misclassifications are especially troubling. Atypical and smaller lesions, which are commonly seen in real clinical practice, were more likely to be incorrectly identified, according to the analysis. We suggest adding more cases of these lesions to the training dataset and investigating the possibility of modifying the loss function to give priority to reducing false negatives in order to address this. Additionally, the durability of the model can be strengthened by incorporating higher-quality images from clinical settings.

### 4.9. Generalizability and Clinical Translation Considerations

The suggested model performs well on the gathered dataset, but further investigation is needed to determine whether it can be applied to larger clinical settings. Due to the dataset’s restriction to two Northern Indian clinics, there may be biases pertaining to patient demographics, imaging procedures, and disease presentation.

**Device variability**: Semi-controlled smartphone-based dermoscopy was used to obtain the images. Dermatoscopes, smartphone cameras, and imaging resolutions vary greatly in practical use. Variations in light, magnification, and color calibration can cause domain shift, which could impair model performance.

**Demographic variability**: Skin cancer manifests differently in people of different ages, genders, and ethnicities.

**Clinical heterogeneity:** There are comparatively obvious diagnostic cases in the sample. However, unclear lesions, early-stage melanoma, and outwardly comparable benign diseases are examples of real-world clinical circumstances that make classification more challenging.

### 4.10. Clinical Translation Roadmap

**External Verification:** To evaluate robustness against domain shift, the model needs to be evaluated on independent multi-center datasets (such as ISIC, HAM10000, and additional Indian clinical datasets).

**Prospective Clinical Evaluation**: To assess diagnostic accuracy, sensitivity, and usefulness in actual clinical processes, a prospective study involving dermatologists should be carried out. It is important to evaluate metrics like decision speed, clinician–AI agreement, and false-negative rates.

**Integration into Clinical Workflow:** Mobile health apps or dermoscopy systems can use the model as a decision-support tool. It is advised to use a triage-based strategy, prioritizing dermatologist review of high-risk lesions identified by AI.

## 5. Conclusions

This study presented a comprehensive deep learning system for the automatic detection of skin cancer using dermoscopic clinical pictures, highlighting a comparative examination of the VGG16 and ResNet-50 architectures. A thorough pipeline was developed, including clinically oriented image preprocessing, lesion segmentation, classification by transfer learning, and the incorporation of explainability. The preprocessing method, namely morphology-based hair removal and lesion-centric segmentation, markedly improved image quality and reduced irrelevant background noise, thus enhancing model performance. The experimental results clearly demonstrate that ResNet-50 outperforms VGG16 in the binary classification of melanoma and non-melanoma skin cancer, achieving an accuracy of 93% and an AUC-ROC of 0.96 compared to VGG16’s accuracy of 89% and AUC-ROC of 0.94. ResNet-50 works better because it has a deeper architecture and residual connections that let it learn more about subtle lesion features, such as texture heterogeneity, color variation, and border anomalies. The study emphasizes the significance of clinical relevance and dependability alongside basic performance metrics.

Owing to the dataset’s limited geographic scope, it only includes photos taken from two clinics in one area, which is one of the study’s main limitations. A bigger, multi-center dataset could enhance the model’s generalizability, even though these photos offer a useful starting point. To better assess the model’s resilience and clinical suitability for a wide range of populations, future research should try to enlarge the dataset to include pictures from other geographical locations and clinical contexts.

Future research should initially transform the binary framework into a multi-class classification system. This would make the system more like dermatologists, who look at more than just two types of lesions. Second, ensemble learning approaches employing architectures that work well with each other should be explored, such as ResNet, DenseNet, and EfficientNet. These ensembles may capture diverse feature representations and enhance generalization, particularly for lesions that are difficult to visualize or differentiate.

## Figures and Tables

**Table 1:** Specification of data collected from clinics.

Subject	Computer Application
**Subject** **Area**	Image Processing, Image Detection, Image Classification
**Types of Data**	Images (.jpeg)
**How the Data Were Acquired**	First, the Ziva Clinic, Hamirpur, was chosen, where these data points were recorded, using smartphone camera images captured after visiting that location on the advice of a dermatologist. Secondly, we collected images from the Sparse Clinic in Ludhiana.
**Data Format**	Raw
**Description of Data Collection**	Samples diagnosed by the dermatologist, with symptoms, class names, sex and infection areas.
**Data Source Location**	Clinics: Ziva Skin & Hair Clinic, Himachal Pradesh; Sparsh Aesthetics, Ludhiana. Country: India.
**Total Dermoscopic Images Collected**	In total, 1200 images. From these images, 1000 images of non-melanoma (NMSC) symptoms or 200 melanoma symptoms were collected.

**Table 2:** Property values after applying traditional methods.

Contrast	Correlation	Energy	Homogeneity	Area	Perimeter	Aspect
197.865951	0.899625	0.983184	0.996957	49,626.5	907.899495	1.0
78.104981	0.646457	0.997699	0.998799	49,729.0	892.000000	1.0
247.332439	0.729723	0.991029	0.996196	49,724.5	894.242641	1.0
118.459221	0.807506	0.994343	0.998178	49,729.0	892.000000	1.0
6650.63914	0.677982	0.768483	0.897723	48,733.5	1042.32901	1.0

**Table 3:** Ablation trials for class weighting for melanoma and non-melanoma.

Loss Class Type	Precision	Recall	F1-Score	AUC
Unweighted Melanoma	0.68	0.75	0.71	0.88
Unweighted Non-Melanoma	0.92	0.94	0.93	0.89
Weighted Melanoma	0.73	0.93	0.82	0.96
Weighted non-Melanoma	0.99	0.93	0.96	0.95

## Data Availability

The image dataset used in this study was collected from clinics and is subject to patient confidentiality agreements. Requests for access to the data can be directed to the corresponding authors, subject to institutional approvals.
